# Do We Have Measures to Reduce Post-operative Cognitive Dysfunction?

**DOI:** 10.3389/fnins.2022.850012

**Published:** 2022-06-02

**Authors:** Thaddee Valdelievre, Zhiyi Zuo

**Affiliations:** Department of Anesthesiology, University of Virginia, Charlottesville, VA, United States

**Keywords:** post-operative complications, post-operative cognitive dysfunction (POCD), predictors, postoperative delirium, enriched environment (EE), cognitive enrichment

## Introduction

In the United States alone, over 50 million general anesthetics for surgery are administered every year (Hall et al., 2017). Reduced cognition post-operatively, referred to here as post-operative cognitive dysfunction (POCD), is decreased neurocognitive function after anesthesia and surgery, as compared to preoperative neurocognitive function. It is commonly measured objectively by a battery of neuropsychological tests (Moller et al., 1998). The nomenclature has since evolved under the term of perioperative neurocognitive disorders, which encompass pre-operative impairment, post-operative delirium (POD), as well as cognitive dysfunction within the first 30 days of surgery, known as delayed neurocognitive recovery, and from days 31 to 12 months known as postoperative neurocognitive disorder (Evered et al., 2018; Evered and Goldstein, 2021). While not as emphasized of a risk to patients as the risk of major bleeding, for example, in current practice, the risk of developing POCD is not negligible. For example, in patients undergoing coronary artery bypass surgery on cardiopulmonary bypass, 53% have evidence of POCD at time of discharge, and 24% still have evidence of POCD 6 months later (Newman et al., 2001). When looking at non-cardiac surgery, a remarkable percentage of patients have evidence of POCD at discharge. An early study published in 1999 looked patients over the age of 60 undergoing a variety of orthopedic, urologic, vascular, and abdominal surgeries. Three months after discharge, approximately 10% of patients had evidence of POCD (Moller et al., 1998). A second study confirmed these findings using young, middle, and elderly age groups, with the young and middle-aged groups having a higher number of intra-abdominal and thoracic surgeries, and a lower number of orthopedic surgeries compared to the elderly group (Monk et al., 2008). More importantly, POCD is associated with poor clinical outcomes. There is a correlation between POCD and mortality (Monk et al., 2008), decreased participation in the labor market (Steinmetz et al., 2009), and time to discharge is longer (Silbert et al., 2006). Given these findings, and with an aging population, research into identifying contributing factors to this neurocognitive dysfunction can have huge impacts on our ability to predict its development, and improve how physicians, patients, and families can prepare, prevent, or lessen the risk and severity of its occurrence.

## Pre-Clinical to Clinical Trials

A variety of basic science mechanisms have been proposed for the development of POCD, including a neuro-inflammatory process *via* oxidative stress and microglial activation, mitochondrial dysfunction, synaptic damage, and damage to the blood brain barrier (Cibelli et al., 2010; Cao et al., 2012). The neuro-inflammatory mechanism is proposed to be the major neuropathological process for developing POCD (Zhang et al., 2015; Lin et al., 2020). This has been substantiated with basic science research using aged rats, with histologic immunostaining showing increased inflammation in the cortex post-operatively (Zhang et al., 2015). Of course, surgical pain and trauma may play a critical role in the development of inflammatory responses in the peripheral tissues and brain (Lai et al., 2021a).

With the mechanisms behind POCD becoming clearer from basic science research, it is logical to consider interventions for patients. Certain risk factors, such as age, education level, physical state, and preexisting neurovascular diseases, have been associated with an increased risk of POCD, but cannot be practically adjusted (Monk et al., 2008; Lin et al., 2020). More readily adjustable contributing factors have been proposed, including type and depth of anesthetic, ambient noise levels, and the surrounding environment (Zhang et al., 2015; Fan et al., 2016; Berger et al., 2018; Lin et al., 2020). Indeed, the most impactful literature to the practicing anesthesiologist would be the comparison between the two primary types of general anesthetic maintenance methods: total intravenous anesthesia (TIVA) with propofol, and inhaled volatile anesthesia, such as sevoflurane (Zhang et al., 2018; Li et al., 2021). While this avenue of research seems to be the furthest along clinically, the lack of definitive evidence suggests that research into other contributing factors, such as ambient noise levels, and the surrounding environment, needs to continue. Of these, clinical data is relatively lacking, or in its infancy, making these non-pharmacological interventions an intriguing avenue of research.

## Type and Depth of Maintenance Anesthetic

A study published in 2011 in the *British Journal of Anesthesia* compared the effects in regards to neurocognitive function of a propofol-based TIVA to a sevoflurane anesthetic in patients undergoing on-pump cardiac surgery (Schoen et al., 2011). The basis of the study stemmed from *in vitro* and pre-clinical trials showing that sevoflurane has neuroprotective properties and can lessen ischemia-reperfusion injury in the event of ischemia (Zhang et al., 2015). The study found no differences between the anesthetic types unless the patient had a cerebral blood oxygen saturation level of <50%. When the levels did decrease to lower than 50%, the patients randomized to the volatile anesthetic group had a lower incidence of cognitive dysfunction (Schoen et al., 2011). While this study only looked at short-term outcomes of 6 days or less, it did provide a link between pre-clinical and clinical trials regarding POCD. To confound the picture, another study of a single center and sub-analysis in 2018 compared the two anesthetic methods in patients aged 65 and older undergoing a variety of cancer surgeries. The study found a higher incidence of POCD in the sevoflurane group compared to propofol-based TIVA one week after surgery (Zhang et al., 2018). Longer-term outcomes, however, were not studied. Most recently, in 2021, a multicenter study was published in *Anesthesiology* in patients 60 years and older undergoing laparoscopic abdominal surgery. Using the definition of POCD put forth by the International Study of Post-operative Cognitive Dysfunction, this study found no differences between the two types of anesthetics 5 to 7 days postoperatively (Li et al., 2021).

Unfortunately, the published studies regarding the differences between the two primary types of anesthesia offer little clarity to this question. While the three studies presented here have mixed conclusions, the differences between the studies, such as patient ages, surgeries, and definition of POCD, make it difficult to compare one study to another. These studies have proposed many more questions than they have answered, laying out a path for future clinical research on POCD, such as using standardized diagnosis criteria for POCD, with a clear stated primary outcome.

Finally, the discussed studies all used processed EEG equipment to monitor the depth of anesthesia with the intention of keeping it similar between groups. Other studies have looked for connections between depth of anesthesia and perioperative neurocognitive disorders, such as POD and POCD. Unfortunately, while processed EEG equipment is certainly much more convenient and simple to use for the practicing anesthesiologist, it is not entirely clear how equivalent it is to interpreting raw EEG wave patterns (Berger et al., 2018). Review of the literature currently does not show clear evidence for routine EEG or processed EEG monitoring during surgery, but clinical trials in this field are expanding and ongoing (Berger et al., 2018; Evered and Goldstein, 2021).

## Effects of Noise

Noise in the hospital environment consistently exceeds the World Health Organization recommendations (Darbyshire and Young, 2013; Scquizzato et al., 2020). Noise has been proposed to increase the risk of POCD by promoting neuro-inflammation and affecting learning and memory (Lin et al., 2020). Lin et al. showed that mice exposed to a noisy environment showed worsened learning and memory compared to controls 1 day after training sessions. Noise increased inflammatory markers interleukin (IL) 1B and IL-6, as well as microglial marker ionized calcium binding adapter molecule 1 (Iba-1) in the hippocampus, which is important in certain types of learning and memory. Further, the group showed that using minocycline, an antibiotic with anti-inflammatory properties, reduced neuro-inflammation and microglial activation and attenuated the learning impairment (Lin et al., 2020). While this study sheds some light on how noise may play a role in promoting neuro-inflammation, which can impact learning, memory, and increase the risk of POCD, it brings up questions that need to be answered. Nevertheless, caution is needed to extrapolate the findings from this animal study to humans. The mice were subjected to a continuous 75 decibel noise level for 6 h a day, and otherwise housed in a quiet environment, not exactly simulating a hospital environment (Darbyshire and Young, 2013). Interestingly, while the group found a difference in learning and memory for “short-term” memory, defined as 1 day after training sessions, there was no difference in long term memory, defined as 8 days after the training sessions, between the control and study groups (Lin et al., 2020). Human clinical trials on noise are likely not practical, nor ethical. Despite this, it may be possible to assess the incidence and relation between POCD, sleep quality, and noise levels in hospital ICUs, wards, and short stay units in both short and long term follow up studies.

## The Enriched Environment

The perioperative enriched environment is an exciting non-pharmacological research avenue into POCD. The enriched environment (EE), essentially described as immersion into surroundings that lead to social, sensory, and cognitive stimulation, has been shown to increase neurogenesis (Fan et al., 2016). Cognitive enrichment removes certain facilitators of increased physical activity to focus more on the cognitive effects of the environment (Gui et al., 2021). Based on pre-clinical trials, both pre-operative and post-operative environmental enrichment may play a role in the development of POCD.

Fan et al. compared the learning and memory performance of mice immersed in an EE after surgery, to mice in a standard environment both with, and without surgery. The group found that surgery decreased neurogenesis, which was attenuated by exposure to an EE. Learning and memory were also impaired with surgery, as expected, but that the mice exposed to the EE had an attenuated impairment. There was no difference in the learning and memory between mice with surgery exposed to the EE and the control mice that did not undergo surgery. These effects, however, were only seen 1 day after training; whereas there was no difference between the groups in “long-term” memory, defined as 8 days after training (Fan et al., 2016). Kawano et al. in 2015 looked into the effects of pre-operative environmental enrichment in both young and aged rats (Kawano et al., 2015). The group found that 2 weeks of an EE attenuated the negative effects of surgery on cognitive dysfunction and neuroinflammatory markers on aged rats, but not on young rats. Gui et al. in 2021 in part looked at the effect of the EE and the cognitively enriched environment on learning and memory in much older, male mice. Exposing the mice to a cognitively enriched environment post-operatively improved learning and memory for both short- and long-term time periods (Gui et al., 2021), which could be clinically promising in patients unable to perform physical activities. Min et al. in 2021 took the preclinical research a step further and looked for the duration of pre-operative environmental enrichment needed to improve learning and memory of surgery mice. The group could not find a protective effect if exposure to the EE was <2 weeks, but noted that at 2 weeks of environmental enrichment, both short and long term memory deficits were attenuated (Min et al., 2021). Recently, Lai et al. used adult and old mice to look at the effects of low, medium, and high intensity exercise regimens on neuroinflammation, learning, and memory leading up to surgery. The group found that low intensity exercise attenuated surgery-induced learning and memory deficits. The group suggests that the mechanism for this protection is mediated *via* exercise-induced restoration of healthy gut microbiota and reduction of valeric acid increased and complement 3 signaling activation caused by surgery. These effects ultimately reduce the impairments in learning and memory (Lai et al., 2021b). Not only did this study reinforce the hypothesis of the effects of an EE, but it also shed light onto the mechanisms of how exercise can attenuate surgery-induced cognitive dysfunction.

These studies certainly have potential, as they provide the groundworks for clinical trials. The recently published Neurobics trial (Humeidan et al., 2021) looking at the effect of cognitive prehabilitation on POD serves as a promising link between laboratory and clinical trials. The study found that using cognitive prehabilitation *via* cognitive exercise software was promising in lowering the risk of POD. Thus, similar clinical studies looking at various effects of both pre-, and post-habilitation exercise regimens as well as the effect of cognitive pre-habilitation and rehabilitation on POCD should be pursued. These measures may be applied in susceptible patients to reduce POCD.

In summary, the research into POCD is looking beyond the differences between maintenance anesthetics, which do not appear to make a significant impact on rates of POCD. EEG interpretation intraoperatively, as well as noise level monitoring, may in future be clinically relevant in the hospital environment pending further studies (Darbyshire and Young, 2013; Berger et al., 2018; Lin et al., 2020; Scquizzato et al., 2020; Evered and Goldstein, 2021). Finally, and perhaps most promising, the EE, socially, physically, and cognitively, has yielded exciting early pre-clinical and clinical studies on its effect with delirium and POCD (Kawano et al., 2015; Gui et al., 2021; Humeidan et al., 2021; Lai et al., 2021b; Min et al., 2021). While future studies involving the EE will undoubtedly require rigorous protocols and follow up, the early research has suggested countless of opportunities for clinical trials and perhaps non-pharmacological interventions to reduce the risk and severity of POCD.

## Author Contributions

ZZ and TV conceived the concept of the paper. TV wrote the draft. ZZ revised it. Both authors contributed to the article and approved the submitted version.

## Conflict of Interest

The authors declare that the research was conducted in the absence of any commercial or financial relationships that could be construed as a potential conflict of interest.

## Publisher's Note

All claims expressed in this article are solely those of the authors and do not necessarily represent those of their affiliated organizations, or those of the publisher, the editors and the reviewers. Any product that may be evaluated in this article, or claim that may be made by its manufacturer, is not guaranteed or endorsed by the publisher.
